# A national stakeholder consensus study of challenges and priorities for clinical learning environments in postgraduate medical education

**DOI:** 10.1186/s12909-017-1065-2

**Published:** 2017-11-22

**Authors:** Caroline Kilty, Anel Wiese, Colm Bergin, Patrick Flood, Na Fu, Mary Horgan, Agnes Higgins, Bridget Maher, Grainne O’Kane, Lucia Prihodova, Dubhfeasa Slattery, Slavi Stoyanov, Deirdre Bennett

**Affiliations:** 1 Medical Education Unit, School of Medicine, University College Cork, Cork, Ireland; 2 Trinity College Dublin, Dublin, Ireland; 3 Dublin City University Business School, Dublin, Ireland; 4 Trinity College Dublin Business School, Dublin, Ireland; 5 School of Medicine, University College Cork, Cork, Ireland; 6 School of Nursing and Midwifery, Trinity College Dublin, Dublin, Ireland; 7 Royal College of Physicians of Ireland, Dublin, Ireland; 8 Children’s University Hospital, Temple St., Dublin, Ireland; 9 Open University of the Netherlands, Heerlen, Netherlands

**Keywords:** Graduate medical education, Postgraduate medical education, Clinical learning environment, European working time directive, Duty hours regulations, Group concept mapping

## Abstract

**Background:**

High quality clinical learning environments (CLE) are critical to postgraduate medical education (PGME). The understaffed and overcrowded environments in which many residents work present a significant challenge to learning. The purpose of this study was to develop a national expert group consensus amongst stakeholders in PGME to; (i) identify important barriers and facilitators of learning in CLEs and (ii) indicate priority areas for improvement. Our objective was to provide information to focus efforts to provide high quality CLEs.

**Methods:**

Group Concept Mapping (GCM) is an integrated mixed methods approach to generating expert group consensus. A multi-disciplinary group of experts were invited to participate in the GCM process via an online platform. Multi-dimensional scaling and hierarchical cluster analysis were used to analyse participant inputs in regard to barriers, facilitators and priorities.

**Results:**

Participants identified facilitators and barriers in ten domains within clinical learning environments. Domains rated most important were those which related to residents’ connection to and engagement with more senior doctors. *Organisation and conditions of work* and *Time to learn with senior doctors during patient care* were rated as the most difficult areas in which to make improvements.

**Conclusions:**

High quality PGME requires that residents engage and connect with senior doctors during patient care, and that they are valued and supported both as learners and service providers. Academic medicine and health service managers must work together to protect these elements of CLEs, which not only shape learning, but impact quality of care and patient safety.

## Background

The *Clinical Learning Environment* (CLE) has been described as ‘*the foundation of graduate medical education’* [1] and refers to the social, cultural and material context in which residents learn while they work [2]. Social theories of learning emphasise the importance of environment for workplace learning [3–7]. Supportive clinical learning environments should afford residents access to supervised participation in patient care, coaching, assessment and feedback, deliberate practice, teamwork, peer collaboration and observable models of practice [8, 9]. Learners’ engagement with the affordances of the CLE leads to acquisition of practical knowledge, skills and attitudes as well as to the development of professional identity [10–17]. Supportive clinical learning environments for postgraduate medical education (PGME) contribute to better patient care through these directs effects on residents and their current and future practice [18, 19]. Regulatory bodies internationally have promoted and mandated the quality assurance of clinical learning environments, signalling their importance [20–23]. High quality clinical learning environments matter because they impact both workplace learning and the quality and safety of clinical care.

Many residents work, learn and develop their professional identities in underfunded [24–26], understaffed [27], uncontrolled and overcrowded clinical environments [28–30]. This presents a significant challenge for learning and for patient safety. The UK General Medical Council (GMC), has stated that ‘*Patient safety is inseparable from a good learning environment and culture that values and supports learners and educators*’ [31]. The GMC annual training survey report 2016 [32] acknowledged the difficulties created by increased demand and short-staffing. One in three supervisors reported that they did not have enough time to fulfil their role. Self-reported heavy workloads in that survey were associated with a greater likelihood of residents feeling forced to work beyond their competence and having patient safety concerns [32]. Intern and resident learning has been reported to decline once a critical level of workload is exceeded [33]. These conditions are associated with doctor burnout [34, 35], lower levels of staff engagement, health and wellbeing [27, 36, 37], all of which negatively impact learning, as well as lower levels of patient satisfaction, poorer standards of patient care [38] and higher mortality [27]. In addition to the issue of excessive workload, the effects of duty hours restrictions, in place in North America and Europe, on learning, patient safety and workload remain uncertain [39–42]. Both the Accreditation Council for Graduate Medical Education (ACGME) [43] and GMC [31] have explicitly linked CLE with quality of patient care and there have been calls for the greater alignment of educational and patient outcomes [44].

Provision of high quality clinical learning environments is critical to the overlapping missions of postgraduate medical education and the wider medical profession. In the UK and the Republic of Ireland, where national health services are under strain, low morale and deep dissatisfaction amongst residents about their working conditions has led to strike action [45, 46]. An exodus of medical graduates and difficulty in recruitment to residencies in both countries [47, 48] send a stark message that change is needed and raise the question; what can academic medicine do to enhance clinical learning environments?

Strategic planning to protect and enhance clinical learning environments is essential in order to mitigate the negative effects of the wider social, economic and political climate. Prioritisation of the most important facilitators and identification of ‘easy wins’ are important steps in this process. The purpose of this study was to develop a national expert group consensus amongst a range of relevant stakeholders; senior doctors, residents, patients, allied healthcare professionals and healthcare managers allowing us to; (i) identify important barriers and facilitators of learning in clinical environments and (ii) indicate priority areas for improvement. Our overarching objective was to provide information to guide policy makers and those tasked with the delivery of graduate medical education in tackling the provision of high quality clinical learning environments in challenging times.

## Methods

Ethical approval for this study was granted by the Clinical Research Ethics Committee of the Cork University Hospitals, Ireland.

### Conceptual orientation

This is an integrated mixed methods study which has been conducted from a pragmatic epistemological stance. This position emphasises the utility of both quantitative and qualitative research approaches to answer a research question, over any epistemological or ontological discordance between them. Within this perspective the interpretative elements of our study have been conducted from a socio-cultural stance, which holds that learning takes place during social interaction in cultural and historical settings [49].

### Setting

This national study was conducted in the Republic of Ireland, which has a comprehensive, government funded public healthcare system. On completion of a medical degree programme graduates spend a year as an intern before becoming fully registered medical practitioners. Those entering hospital specialties then spend 6–8 years in training programmes which are similar to UK Foundation, Core and Specialist training and broadly analogous to North American residencies and fellowships. A variety of terms are used, both internationally and nationally, to describe doctors in postgraduate medical education, these include; junior doctors, trainees, doctors-in-training, non-consultant hospital doctors and residents. Similarly, terms to describe senior doctors include consultants, attending physicians, supervisors and trainers. The latter two terms suggesting a formal educational role. In this paper we will use the terms resident and senior doctor unless directly quoting participants.

This study is part of a larger project funded by the Health Research Board, Ireland. The research team is a multi-disciplinary group including residents, senior doctors, medical educators, experts in organisational behaviour, and representatives of nursing and allied healthcare professions as well as patient representation.

### Recruitment and participation

We issued an invitation via email to experts and stakeholders in postgraduate medical education nationally to participate in a consensus building process. We purposively selected attending physicians, residents, health service managers, allied healthcare professionals and patient representatives, on the basis of their expertise and experience of clinical learning environments. Doctors with senior roles in postgraduate medical education were targeted, as were clinicians who supervise trainees daily in the workplace. A range of allied healthcare professionals were targeted in a similar manner. Each of the stakeholder groups included interfaces with postgraduate training ‘on the ground’ in the clinical environment and/or contributes to the policy and structures which govern training. We hypothesised that they might provide unique perspectives in respect of our research questions.

### Data collection

Group Concept Mapping (GCM) is a novel integrated mixed method approach to identify an expert group’s understanding about a topic. GCM builds on the strengths of other structured consensus building methods, such the Delphi method, mitigating some of their weaknesses [50]. The aim of GCM is to depict unique ideas on a particular topic by converting complex qualitative data into conceptual maps [51] showing how individual ideas are related to each other, and generating rating data relating to the relative importance of an idea, and its perceived achievability [51].

GCM consists of five phases:

**Phase 1: Brainstorming & Pruning**: We provided participants with a web-based link to an online platform for data collection [52]. Participants completed a short demographic questionnaire and identified facilitators and/or barriers to learning in clinical environments in response to the following prompt:

*One specific barrier or facilitator to trainee doctors learning within the clinical environment is...’*

Participants could provide as many statements as they wished and we asked for a minimum of five from each. The second step was idea pruning, a data cleaning process undertaken by the research team. We reviewed the initial list of statements for any repetition, ambiguity or more than one idea. We deleted, re-worded or split statements as required.

**Phases 2 & 3: Sorting & Rating:** In keeping with the GCM method, a subgroup of the original participants was selected to sort the edited list of statements into groups, based on similarity of the ideas therein. These participants were then asked to rate each statement for *importance* using the following prompt;

*‘Rate the relative importance of each statement as a facilitator or a barrier to trainee doctors’ learning within the clinical environment using a scale ranging from 1 (relatively unimportant) to 5 (extremely important).’*

The process was then repeated for each statement regarding *‘ease to address’*.

**Phase 4**: **Data Analysis:** Concept System Global software was used for the quantitative data analysis. Multi-dimensional scaling produced a point map of the statements based on the principle that statements which participants had more frequently grouped together during the sorting process on the basis of their similarity, appear close to each other on the point map [53]. A bridging value was calculated for each statement. This is a statistic ranging from 0 to 1 which indicates how often a statement is grouped with others adjacent to it on the concept map, and whether participants have grouped it with others further away.

Hierarchical cluster analysis embedded in GCM initially treats each individual idea as a separate cluster, and continues to merge ideas until it arrives at one cluster [54]. Mean bridging value for a cluster is calculated on the individual bridging values of statements composing that cluster [55]. A lower bridging value indicates that a statement has greater cohesion with others in that cluster. Lower mean bridging value for a cluster corresponds to a greater level of agreement on the content of that cluster [55].

Mean rating scores for importance and ease to address for each statement and cluster were calculated. The clusters and ratings produced by doctors were compared with those produced by the other participants.

**Phase 5: Interpretation of the results:** The GCM software generated an initial cluster solution and offered a sequence of cluster merges which could be undertaken if there was sufficient conceptual similarity between clusters to do so. This qualitative element of the mixed methods analysis is underpinned by researcher interpretation and judgement [53]. DB, AW and CK co-constructed a thematic interpretation of the statements in each cluster during a series of meetings. Judgements to merge clusters or not were based on similarity of meaning of the statements therein. Once merges were agreed, DB, AW and CK proposed meaningful labels for the final clusters, informed by our orientation towards social theories of learning e.g. Communities of Practice. These were labels were debated and finalised with the wider multi-professional research team.

## Results

Fifty-five participants contributed to the first phase of the GCM, brainstorming. All stakeholder groups were represented. Sixty-five percent of participants were female. Table 1 shows their distribution by category.

**Table 1:** Participants by category

Participant Category	n	%
Resident	10	18%
Senior doctor / supervisor	9	16%
Senior Strategic Role in PGMET	10	18%
Patient Representative	4	7%
Allied Healthcare Professional	13	24%
Health Services Management	9	16%
Total	55	100%

Two hundred and six statements relating to facilitators and barriers to learning in clinical environments were generated. Following pruning, 97 unique ideas remained; 78 were barriers to learning and 19 were facilitators. Twenty-seven participants contributed to the sorting and rating phase.

**Fig. 1:**
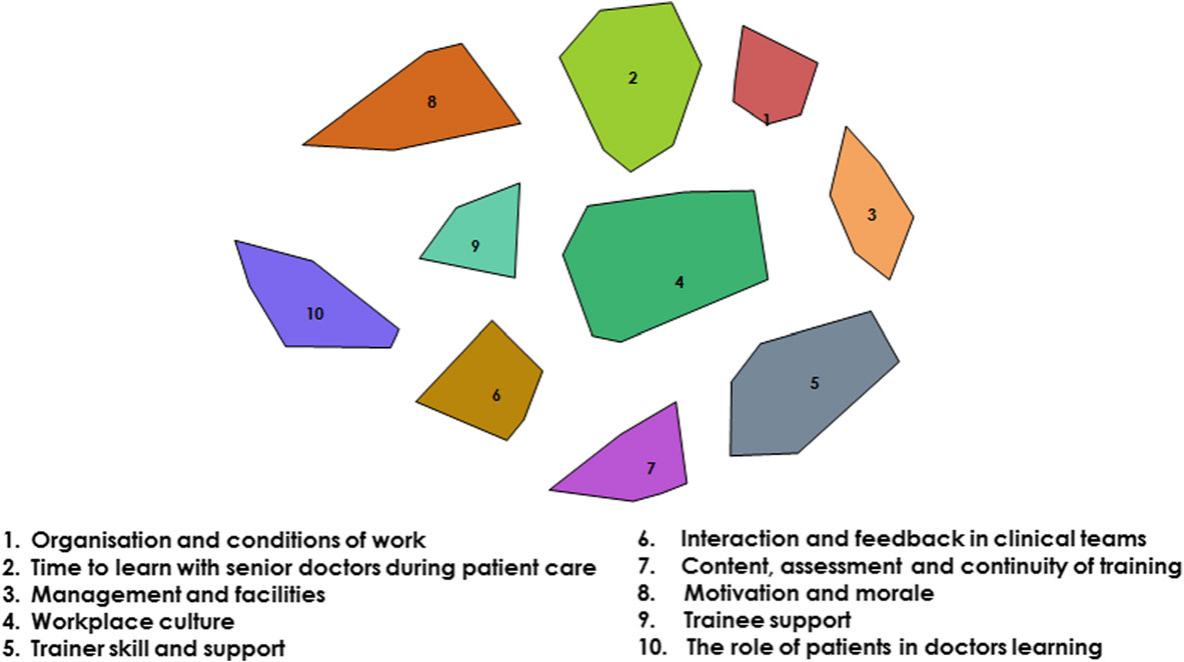
10 cluster solution with domain names

Following review as outlined above, a 10 cluster solution describing key domains of clinical learning environments, shown in Fig. 1, was determined. This decision was based on the conceptual sense of merging clusters based on the themes of the statements within them.

Clusters were named as shown in Table 2 and Fig. 1. Mean cluster bridging values (BV), which are an indicator of the coherence of the cluster, are shown in Table 2. The lower the bridging value the more cohesive the cluster. Sample statements from each cluster are also presented.

**Table 2:** Ten clusters with names, definitions and sample statements for each

	Title and Definition	Mean BV	Sample Statement	BV
1	Organisation and conditions of work – *relating to the tension between providing service in busy environments and needing time to reflect and learn*	0.19	Barrier: Clinical areas are too busy and this acts as a barrier to residents’ learning.	0.05
2	Time to learn with senior doctors during patient care - *relating to the way that residents learn from work alongside senior doctors as they follow the patient pathway*	0.23	Barrier: Time pressure at work has meant that the mentorship/apprenticeship role is lost and residents no longer have the time/opportunity to discuss a case in-depth with a Senior Doctor.	0.11
3	Management and facilities - *relating to the way in which hospital management values and facilitates training and the provision of facilities to support training at hospital sites*	0.46	Barrier: A lack of commitment by hospital management teams to training. Management support for the training element of the workplace is inadequate - seen as very secondary to workload.	0.4
4	Workplace culture - *referring to the way in which learning and residents are valued in the workplace*	0.23	Facilitator: Culture of the clinical site values residents, listens to their views and takes appropriate action in response.	0.07
5	Trainer (Senior doctor) skill and support - *referring to who does the training and how they are supported*	0.39	Barrier: There is an unwillingness to accept that education and training programmes can be delivered by people other than full time attendings.	0.2
6	Interaction and feedback in clinical teams- *relating to team dynamics including the provision of feedback to the resident while working together*	0.55	Facilitator: Residents learn best when they are challenged to state what they should do with regard to patient management and are affirmed and supported in their choices.	0.39
7	Content, assessment and continuity of training - *relating to learning and assessment rooted in clinical practice with effective communication between senior doctors about performance*	0.48	Barrier: Poor communication between supervisors for different clinical placements.	0.46
8	Motivation and morale- *relating to morale within the healthcare system and its impact on the motivation and attitude of learners and other staff*	0.75	Barrier: Low morale amongst all staff as they are over worked and leading to stress and tense staff.	0.41
9	Resident support - *referring to reception of the doctor-in-training into team, collegiality, respect and support to work within scope of practice and to challenge constructively.*	0.36	Facilitator: The resident is encouraged to work within his/her scope of practice to safely develop skills under supervision.	0.34
10	The role of patients in doctors’ learning *- referring to patient expectations of care, willingness and provision of feedback*	0.44	Facilitator: Patients more informed in relation to care provision and willing to challenge those delivering care.	0.22

Figure 1 shows the relationship of the clusters to each other; proximity representing domains which are more closely related to each other and distance vice versa. *The role of patients in doctors’ learning* can be seen to be relatively distant from the remaining 9 clusters indicating that it is conceptually more distinct. *Work place culture* is at the centre of the map and is immediately adjacent to 7 of the 9 remaining clusters which suggests that most key aspects of clinical learning environments are linked to culture. *Time to learn with senior doctors during patient care* is relatively distant from *Content, assessment and continuity of training* and *Trainer (Senior doctor) skill and support* and these appear to represent two distinct aspects of clinical learning environments; the informal learning that happens while delivering patient care, and way in which that learning is structured, organised and resourced as part of a residency programme.

### Rating

All clusters were rated as important to address, with mean ratings ranging from 3.42 to 3.96 on a scale from 1 to 5. There was a wider spread of mean ratings of clusters on ease to address (2.37–3.68). We compared the ratings provided by doctors (*n* = 19) and non-doctors (*n* = 8) and found that these were highly correlated, *r* = 0.7 for importance and *r* = 0.99 for ease to address. The non-doctor group rated *Time to learn with senior doctors during patient care* and *Organisation and conditions of work* as relatively less important, third and sixth out of ten respectively, compared with doctors, who rated these the top two most important domains. Both groups rated *The role of patients in doctors’ training, Content, assessment and continuity of training*, and *Trainer (senior doctor) skill and support* as least important, in that order Table 3.

**Table 3:** Cluster ratings for *Importance* and *Ease to Address*

Most to least	Importance (5 = Very Important)	Mean cluster rating importance	Ease to Address (5 = Very Easy)	Mean cluster rating ease to address
1	Cluster 9: Resident support	3.96	Cluster 7: Content, assessment and continuity of training	3.68
2	Cluster 2: Time to learn with senior doctors during patient care	3.90	Cluster 5: Trainer skill and support	3.56
3	Cluster 6: Interaction and feedback in clinical teams	3.89	Cluster 10: The role of patients in doctors’ training	3.35
4	Cluster 1: Organisation & conditions of work	3.81	Cluster 3: Management & facilities	3.20
5	Cluster 3: Management & facilities	3.73	Cluster 9: Resident support	3.19
6	Cluster 4: Workplace culture	3.72	Cluster 4: Workplace culture	3.01
7	Cluster 8: Motivation and morale	3.63	Cluster 6: Interaction and feedback in clinical teams	3.01
8	Cluster 10: The role of patients in doctors’ training	3.59	Cluster 8: Motivation and morale	2.55
9	Cluster 5: Trainer skill and support	3.51	Cluster 1: Organisation and Conditions of Work	2.51
10	Cluster 7: Content, assessment and continuity of training	3.42	Cluster 2: Time to learn with senior doctors during patient care	2.37

### Individual statements

Ratings for individual statements identified some very specific areas which participants viewed both important and relatively easy to address. Feedback was most prominent amongst these;

*Gaining good regular feedback on their performance by those in the immediate clinical environment is a facilitator to learning (Importance 4.56, Ease to address 4.33).*

*Patient feedback to the young doctor is beneficial and should be encouraged, especially in how they have interacted with the patient (Importance 4.12, Ease to address 4.04).*

Protected time for teaching and learning was a second prominent theme;

*Protected time being allocated to both senior doctors and residents to facilitate tutorials (Importance 4.0, Ease to address 3.42).*

*Bleep free educational sessions are still aspirational in most hospitals (Importance 3.8, Ease to address 3.54).*

## Discussion

At the outset of this study we aimed to provide information to guide policy makers and those tasked with the delivery of postgraduate medical education in tackling the provision of high quality clinical learning environments in challenging times. We have done so by reporting stakeholders’ consensus on the most important domains in CLEs, and the most important barriers/facilitators to learning within them. We have also reported stakeholders’ perceptions of the relative ease with which these issues can be addressed. These findings can contribute to targeted improvements to clinical learning environments.

### Principal findings

In this study stakeholders identified ten distinct domains within clinical learning environments. These domains were mapped to provide a visual representation of their relationships. *Workplace culture* lay in a central position on the cluster map (Fig. 1), directly adjacent to 7 of the other clusters, suggesting that most aspects of clinical learning environments are culturally embedded. There was consensus amongst doctors and other participants that all of the domains identified are important to address to enhance resident learning; however, some domains were rated more important than others.

Domains rated most important were those which related to residents’ connection to and engagement with more senior doctors and other members of the clinical team (*Resident Support, Time to Learn with Senior Doctors during Patient Care and Interaction and Feedback in Clinical Teams*). This is in keeping with social learning theory, for example Communities of Practice theory [3], which emphasises the importance for newcomers to a community to learn by working alongside more senior members. Learning, through observation, role modelling, discussion and feedback all takes place in this context. Participants identified shorter working hours, subsequent to the implementation of the European Working Time Directive (EWTD), as disrupting learning through a reduction in time spent with senior doctors and a disintegration of clinical teams. Under the EWTD residents work no more than 48 h per week in total. Participant statements indicated that less time spent in the clinical environment reduces opportunities to learn through clinical work, to benefit from mentorship and to follow the patient pathway, compounding the challenges of learning in a healthcare system under strain. These perspectives are consistent with some of the literature examining the effects of restricted duty hours [56]; however, published data on the impact of EWTD on training has been mixed [40], perhaps due to the influence of local differences in how the regulations are implemented. In keeping with the GMC national training survey report [57] our findings suggest that poor rota design and scheduling has a negative impact on learning.

*Organisation and conditions of work* was a strongly coherent and relatively more important domain also identified by participants. Barriers in this domain referred to busyness, service pressure and overcrowding. This emphasises the dual purpose of clinical environments; supporting both patient care and learning, and confirms that service pressures impact opportunities to learn, resulting in cognitive overload, limiting time to reflect and discuss [33], and through constraints on physical space.

Domains relating to the structure and organisation of residency programmes, such as *Content, assessment and continuity of training*, and *Trainer (senior doctor) skill and support* were viewed as somewhat less important. When mapped (Fig. 1), it was also apparent that these domains, and that of *Management and facilities*, were grouped together, distant from *Time to learn with senior doctors during patient care, Trainee support* and *Motivation and morale.* The former group of domains can be conceptualised as the imprint of training programmes and management, on the historically, socially and culturally constructed practice of patient care; the formal versus the informal aspects of postgraduate training. Participants identified these formal areas as relatively easy to address, in keeping with fact that improvements and innovations in postgraduate training typically involve changes to content, format and organisation [58–60]. The impact of such changes on day to day learning and supervision has been questioned [59, 61]. Dijkstra et al. [13] found the influence of structural changes on trainees’ learning was mediated by ‘on the ground’ features of the clinical learning environment. It has been suggested that local approaches to cultural change may be effective [59, 62] in improving clinical learning environments. Our findings suggest that our participants recognised a separation between daily practice and externally determined format and organisation. Programme directors and policymakers need to recognise the limitations of approaches which focus on the latter.

*Organisation and conditions of work* and *Time to learn with senior doctors during patient care* were rated as the most difficult areas in which to make improvements. The consensus amongst our participants the combination of heavy clinical workload and duty hour restrictions would be challenging to address may arise from the fact that both are seen as beyond the sphere of influence of academic medicine and healthcare management, but rather the consequences of national economic recession and European legislation. None the less, it is clear that regulators expect those ‘on the ground’ to deal with these challenges. The General Medical Council (UK) places responsibility for adequate staffing, workload and rota design to support learning on Local Education Providers within the NHS [31] and the Medical Council of Ireland has identified the need for an integrated approach at clinical sites, joining up corporate areas within the health service with responsibility for patient safety and quality of clinical care and those responsible for management of the clinical learning environment [63].

*Trainee Support* was identified as the most important area to address. This domain refers to reception into the clinical team, collegiality, respect and support to work within scope of practice and to challenge constructively*.* Participants rated this aspect as moderately easy to address, perhaps because it is within the control of individuals to be welcoming, supportive and respectful of residents. Bullying, and rude, dismissive and aggressive communication are features of healthcare environments internationally [64]. Workload and workplace stress have been shown to trigger such behaviour. Junior members of staff are more likely to be on the receiving end of such communication and it has a negative impact on mental health [64, 65].

### Implications for practice

The link between clinical learning environments and the quality and safety of patient care is a strong driver for stakeholders in postgraduate medical education and health service delivery to work together to strengthen both. Our findings point to the relatively greater importance of the informal over formal aspects of workplace learning and a need to prioritise the preservation of residents’ opportunities to learn and work alongside senior doctors when service delivery is being planned and structured. There is some evidence that the manner in which restricted duty hours are implemented can mitigate the impact on learning [66]. It has been suggested that affording training programmes flexibility [67] and undertaking precise workload analysis might be helpful [66]. Such an approach would enable learning to be taken into account and optimised in the implementation of compliant rotas and schedules. This approach was recommended in Sir John Temple’s 2010 report into the impact of EWTD in the UK [56]; however, there is very little published literature describing innovative ways to comply with duty hours regulations while maximising learning. Further research should focus on exploring the implementation of duty hours’ regulations in the full range of training contexts.

A continual focus on residents as learners, in need of and deserving of support from all in the healthcare environment, is essential. Faculty development has been flagged as a key element of high quality postgraduate medical education and provides a means to enhance the value placed on learning and learners in clinical environments [59]. It could also contribute to the ‘easy win’ identified by participants of ensuring that resident receive more high quality feedback. Culturally embedded negative behaviours towards residents present a significant challenge. Illing et al. [68] make recommendations arising from their review of the literature on bullying which emphasise the importance of senior leadership in initiating culture change. Exploration of other means to generate cultural change with meaningful impact on CLEs and the learning experience of residents is needed [62].

### Strengths and limitations

A strength of this study is that it is a national study which included a wide range of stakeholders in postgraduate medical education and used a rigorous methodological approach to describe their consensus. These are individuals at the frontline of medical training as well as those with more strategic roles; therefore, our findings are rooted in both practice and policy. A limitation of the study is that it was undertaken in the context of the Irish healthcare system and may not be fully transferable to other contexts.

## Conclusions

High quality graduate medical education requires that residents have time to engage and connect with senior doctors during patient care, and that they are valued and supported as both learners and service providers. Academic medicine and health service managers must work together to protect these key elements of clinical learning environments which not only shape learning, but impact patient safety and the quality of patient care.

## Abbreviations

ACGME: Accreditation Council for Graduate Medical Education
CLE: Clinical Learning Environment
EWTD: European Working Time Directive
GCM: Group Concept Mapping
GME: General Medical Council
NHS: National Health Service
PGME: Postgraduate Medical Education
